# Author’s Response to Letter to the Editor: “A Deep Dive into Japan’s State of Emergency: How Political Decisions Affected Post-COVID-19 Syndrome”

**DOI:** 10.31662/jmaj.2024-0026

**Published:** 2024-07-08

**Authors:** Yasuha Kinugasa, Mara Anais Llamas-Covarrubias, Katsuhiko Ozaki, Yoshiaki Fujimura, Takeki Ohashi, Kou Fukuda, Shinichi Higashiue, Yusuke Nakamura, Yumiko Imai

**Affiliations:** 1 National Institutes of Biomedical Innovation, Health and Nutrition (NIBIOHN), Ibaraki, Japan; 2 Tokushukai Information System, Osaka, Japan; 3 Tokushukai Group, Osaka, Japan

**Keywords:** Post-COVID-19 Syndrome, Long COVID, SARS-CoV2

We thank Dr. Kaneda for providing valuable comments ^(1)^ on our study entitled “Post-coronavirus disease 2019 syndrome in Japan: An observational study using a medical database” in which we reported on the incidence of this syndrome in Japan ^(2)^. We appreciate the opportunity to provide responses and further discussion.

We agree with the importance of elucidating the impact of various social determinants on the incidence of post-COVID-19 syndrome during the early stages of the pandemic. Our study included patient data from all over Japan, from Hokkaido to Okinawa. In particular, more than 60% of the patients in our study were in Specific Alert Areas A, namely, Tokyo, Kanagawa, Saitama, Chiba, Osaka, Hyogo, and Fukuoka prefectures, in which an emergency declaration was issued in the early stages of the pandemic. As a previous study showed, the emergency declaration affected people’s mobility and access to medical care ^(3), (4)^. Lack of exercise due to refraining from going around may have affected health conditions, resulting in an increased incidence of post-COVID-19 syndrome. In particular, moderate exercise can reduce the incidence of depression ^(5)^, which occurred at a high incidence as a post-COVID-19 symptom among older adults in our study; therefore, mobility restriction under the emergency declaration may have increased the rate of depression. Furthermore, various medical care provisions may have been restricted under the emergency declaration, including the postponement or cancellation of elective surgeries. These restrictions may have affected the incidence of post-COVID-19 syndrome. For instance, restricting treatment for patients in the early stages of COVID-19 can lead to other symptoms. This type of medical care restriction may have had a major effect on the incidence of post-COVID-19 syndrome and on medical care in the later stages of the pandemic because of the strain on the healthcare system.

Given these points, we would like to engage in further analysis of the relationship between the incidence of post-COVID-19 symptoms and social determinants, such as people’s behavior and the strain on healthcare systems, by classifying the political state of the area during the early pandemic phase and changes in the social environment to provide further insight into post-COVID-19 symptoms.

## Article Information

### Conflicts of Interest

None
